# *Paracoccidioides brasilinsis*-Induced Migration of Dendritic Cells and Subsequent T-Cell Activation in the Lung-Draining Lymph Nodes

**DOI:** 10.1371/journal.pone.0019690

**Published:** 2011-05-18

**Authors:** Suelen Silvana dos Santos, Karen Spadari Ferreira, Sandro Rogerio Almeida

**Affiliations:** 1 Departamento de Análises Clínicas e Toxicológicas, Faculdade de Ciências Farmacêuticas, Universidade de São Paulo, Brasil; 2 Departamento de Ciências Biológicas, Universidade Federal de São Paulo, São Paulo, Brasil; Instituto Butantan, Brazil

## Abstract

Paracoccidioidomycosis is a mycotic disease caused by a dimorphic fungus, *Paracoccidioides brasiliensis (Pb)*, that starts with inhalation of the fungus; thus, lung cells such as DC are part of the first line of defense against this microorganism. Migration of DC to the lymph nodes is the first step in initiating T cell responses. The mechanisms involved in resistance to *Pb* infection are poorly understood, but it is likely that DC play a pivotal role in the induction of effector T cells that control *Pb* infection. In this study, we showed that after *Pb* Infection, an important modification of lung DC receptor expression occurred. We observed an increased expression of CCR7 and CD103 on lung DC after infection, as well as MHC-II. After *Pb* infection, bone marrow-derived DC as well lung DC, migrate to lymph nodes. Migration of lung DC could represent an important mechanism of pathogenesis during PCM infection. In resume our data showed that *Pb* induced DC migration. Furthermore, we demonstrated that bone marrow-derived DC stimulated by *Pb* migrate to the lymph nodes and activate a T helper (Th) response. To the best of our knowledge, this is the first reported data showing that *Pb* induces migration of DC and activate a T helper (Th) response.

## Introduction

Paracoccidioidomycosis (PCM) is a mycotic disease caused by a dimorphic fungus, *Paracoccidioides brasiliensis* (*Pb*), which initiates a deep mycosis that primarily attacks lung tissue. PCM is the most prevalent deep mycosis in Latin America. Epidemiological and experimental evidence suggests that natural infection is initiated after inhalation of the conidia produced by the mycelial form of the fungus [1]. Clinical forms of PCM vary from a localized and benign disease to a progressive and potentially lethal systemic infection [2]. The broad spectrum of clinical forms is a result of the influence of several factors on disease severity including host response and virulence of the infecting agent [3]. The acute or severe form of the disease is associated with deficient cell immunity, high antibodies (Abs) levels, and preferential secretion of type 2 cytokines [4].

Dendritic cells (DC) are antigen-presenting cells that act as sentinels in peripheral tissues, constantly sampling the antigens in their environment. Lung DC play a pivotal role in infections caused by airborne pathogens such as *Mycobacterium tuberculosis*, *Aspergillus fumigatus* and *Cryptococcus neoformans* [5] [6] [7] [8]. The DC population lining the lungs is key to the initiation of T cell responses after pulmonary challenge [9]. DC remain quiescent until activated, at which point they migrate to the draining lymph nodes (DLN) [10], present antigen, and initiate T cell activation [11].

The mechanisms involved in resistance to *Pb* infection are poorly understood, but it is likely that DC play a pivotal role in the induction of effector T cells that control *Pb* infection. For example, the production of IFN-γ and IL-10, both effector T cell products, appears to be involved in the resolution and dissemination of PCM in both mice and humans [12] [13].

Because DC are the most effective antigen presenting cells (APC) for inducing cell-mediated immune responses, it is important to investigate lung DC and their potential for lymph node migration and immune response initiation. In this study, we analyzed the role that DC played in modulating *Pb* infection. To the best of our knowledge, this is the first reported data showing that *Pb* induces migration of DC. Furthermore, we demonstrated that bone marrow-derived DC stimulated by *Pb* migrate to the lymph nodes and activate a T-cell response.

## Methods

### Ethics Statement

The protocol was approved by the Committee on the Ethics of Animal Experiments of the University of Sao Paulo in 02/2009 (Permit Number: 154).

### Animals

For this study, we used 8 to 12-week-old BALB/c female mice obtained from the specific pathogen free facility of the University of Sao Paulo and kept in the animal room, with constant temperature and humidity with cycle of dark/light, conformed to institutional guidelines for animal care and welfare.

### P. brasiliensis strains

The yeast form of the highly virulent *P. brasiliensis* strain 18 was grown in Sabouraud-agar. The strain used was recovered from animals before the experiments. A suspension of fungi was prepered with sterile PBS and yeast cells were adjusted to 1×10^5^ cells in 50 µl, based on hemocytometer counts. Viability was determined with Janus Green B vital dye (Merck) and was always higher than 90%.

### Mouse infection

Mice were challenged with an intratracheal inoculation of 1×10^5^ yeast cells of the virulent *Pb* strain 18. After 12 or 24 hrs, the lungs and lymph nodes were removed prior to DC analysis.

### Analysis of lung and lymph node DC phenotype after *Pb* infection

The effects of *Pb* on surface molecule expression in lung DC were investigated after intra-tracheal (i.t.) infection at 12 and 24 h. Cells were isolated from lungs as previously described [14]. After perfusing the pulmonary vasculature with 5 ml of PBS containing 100 U/ml heparin, the lungs were minced and incubated for 90 min at 37°C in digestion buffer containing 0.7 mg/ml collagenase IV (Sigma-Aldrich- St. Louis-USA) and 30 mg/ml type IV bovine pancreatic DNase I (Sigma-Aldrich- St. Louis-USA). Large particulate matter was removed by passing the cell suspension through a small loose nylon wool plug. The mediastinal and axillary lymph nodes were removed and disrupted, and the cells were analyzed. DC phenotype was determined by flow cytometry using a FACSCantoII (Becton Dickinson). In order to determine the expression of class II MHC co-stimulatory and adhesion molecules, we used labeled Mabs against mouse PE-CD11c (HL3) PECy5-CD11c (HL3), FITC-CD11c (HL3), PE-MHC-II (M5-114.15.2), PE-CD80 (16-10A1), FITC-CD86 (GL1), PE-CCR7 (4B12), APC-DC-SIGN (LWC06), and APC-CD103 (2E7) (All antibodies were obtained from BD Biosciences, San Jose, CA). FlowJo was used for analysis of flow cytometry data. To distinguish autofluorescent cells from cells expressing low levels of individual surface markers, we established upper thresholds for autofluorescence by staining samples with fluorescence-minus-one (FMO) control stain sets [15]. In these sets, a reagent for a channel of interest is omitted.

Bone marrow-derived DCs were generated according to described methods [16]. Femurs and tibias were flushed with 3–5 ml of PBS in 1% BSA. Bone marrow cells were differentiated into DC by culturing in RPMI supplemented with 10% Fetal Calf Serum (FCS), 10 mg/ml gentamicin, and recombinant cytokine GM-CSF (50 ng/ml) for 7 days. On days 3 and 5, the nonadherent cells (granulocytes and lymphocytes) were removed, and the media was replaced with fresh media and growth factor. On day 7, the nonadherent cells were removed and analyzed by FACS using DC cell surface markers. The bone marrow-derived DCs expressed MHC class II, CD80, CD40, CD11b and CD11c (data not shown).

### Analysis of *in vivo* DC migration

In order to analyze DC migration capability following *Pb* interaction, we first used Bone-marrow derived dendritic cell (BM-DC) marked with CFSE. BM-DCs were labeled for 20 min at 37°C with 5 µM carboxyfluorescein diacetate succinimidyl ester (CFSE) (Molecular Probes, Eugene, OR). Following incubation, labeling was stopped by the addition of RPMI/10% FBS, and the cells were washed 3 times with RPMI at room temperature. To reduce the amount of unbound CFSE in cell suspensions, the cells were incubated at 37°C for 5 min after the second wash and prior to the third wash. The CFSE-labeled DCs were incubated with *Pb* (1∶1) for 4 hrs and injected intratracheally (1×10^5^ DC in 50 µl PBS). After 12 and 24 hrs, the mediastinal and axillary lymph nodes were removed. Cells were isolated and analyzed by flow cytometry as described above. As a control, we used CFSE-labeled BM-DC without incubation with *Pb*.

### CD4 T-cell activation after *Pb*-pulsed DC administration

To determine the type of T-cell activation occurring after administration of *Pb*-pulsed DC, BM-DC (1×10^5^) were incubated with *Pb* (1∶1) for 4 hrs and injected intratracheally. After 5 days, the mediastinal and axillary lymph nodes were removed and disrupted, and the cells were analyzed. After surface staining for CD4, the lymph node cells were fixed, permeabilized, and stained by anti-IL-10, anti-IL-4 and anti-IFN-γ Abs (BD). The cells were analyzed by cytometry as described above.

### CFSE labeling of migrating DC

To detect migrating lung DC, mice were lightly anesthetized and intratracheally administered 1×10^5^ *Pb* diluted in PBS and 50 µl 8 mM CFSE (Fluka, Buchs, Switzerland) diluted in PBS. This method is based on the labeling of intracellular proteins and provides stable labels for at least 8 weeks in non-dividing lymphocytes. This labeling procedure has frequently been used to stain cells in the lungs and has allowed the tracking of respiratory DC migration. After 12 and 24 hrs, the mediastinal and axillary lymph nodes were removed. Cells were isolated and analyzed by flow cytometry as described above. Mice administered only CFSE were used as controls.

### DC transport yeast form of *Pb* to draining lymph nodes

#### Labeling of yeast with FITC

Live yeast were suspended in 0.1 M carbonate buffer (pH 9.3) at 2×10^8^/ml and added to 200 µl of FITC in DMSO, as described [17]. After incubation for 2 h at room temperature while being protected from light, the suspensions were diluted and washed twice in PBS (pH 7.2) to remove all detectable free-FITC as determined by fluorescence measurement of the supernatants when compared to PBS alone. The yeast pellets were then counted and diluted in PBS to the desired concentration. FITC labeling did not affect the viability of cells. After labeling, 1×10^6^ *Pb* were injected intratracheally. After 12 h, the lymph node cells were removed and analyzed by cytometry as described above. Animals injected with FITC only were used as controls.

### Statistics

Statistical comparisons were made by analysis of variance (ANOVA) and by Tukey-Kramer test. All values were reported as the mean +/− standard error of the means.

## Results

### Pb infection alters the number and phenotype of lung DCs

**Figure 1:**
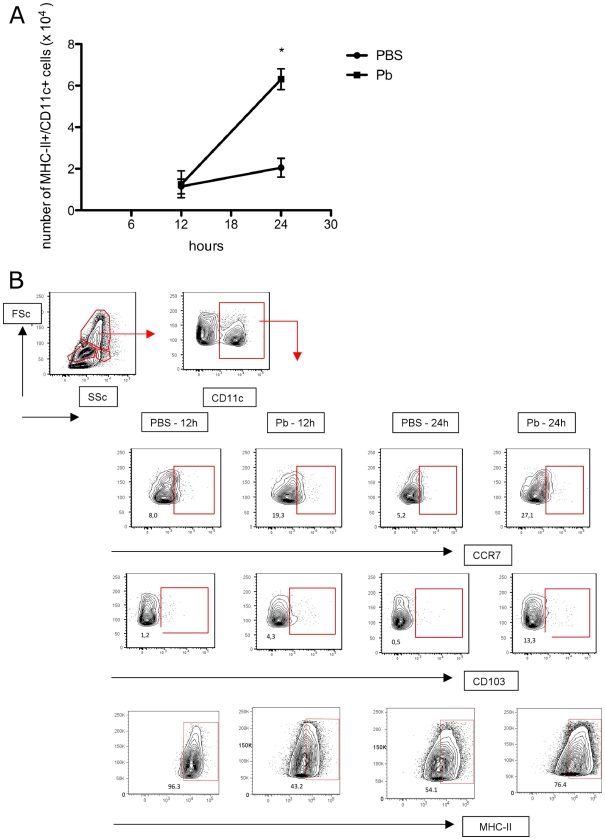
Migration and phenotype of DC in lung during Pb infection. A- At several time points after Pb infection, the absolute numbers of DC (MHC-II+/CD11c+) were determined in the lung. Error bars represent the SEM. B-Phenotype of lung DC after Pb infection, the gate represent positive cells, determined by FMO, as described in the M&M. This figure is representative of 3 different experiments. The numbers represent the percentage of positive cells. The experiment was performed at least 3 times. *p<0.05 when compared with PBS treatment.

To study the role of *Pb* infection on lung DC, we infected BALB/c mice intratracheally (i.t.) with the yeast form of *Pb*. At 12 and 24 hrs after infection, we analyzed the number and phenotypes of lung DC by flow cytometry. We showed an increased in the number of MHC-II^+^/CD11c^+^ cells (DC) after 24 hrs of *Pb* infection (Figure 1A). In addition, an increase of DC expression of CD103, MHC-II, and CCR7 when compared with the controls (PBS) was observed (Figure 1B). No significant differences were observed in the expression of CD80, CD86 and DC-SIGN (data not shown).

### In vivo DC Migration to the lung-draining lymph nodes

**Figure 2:**
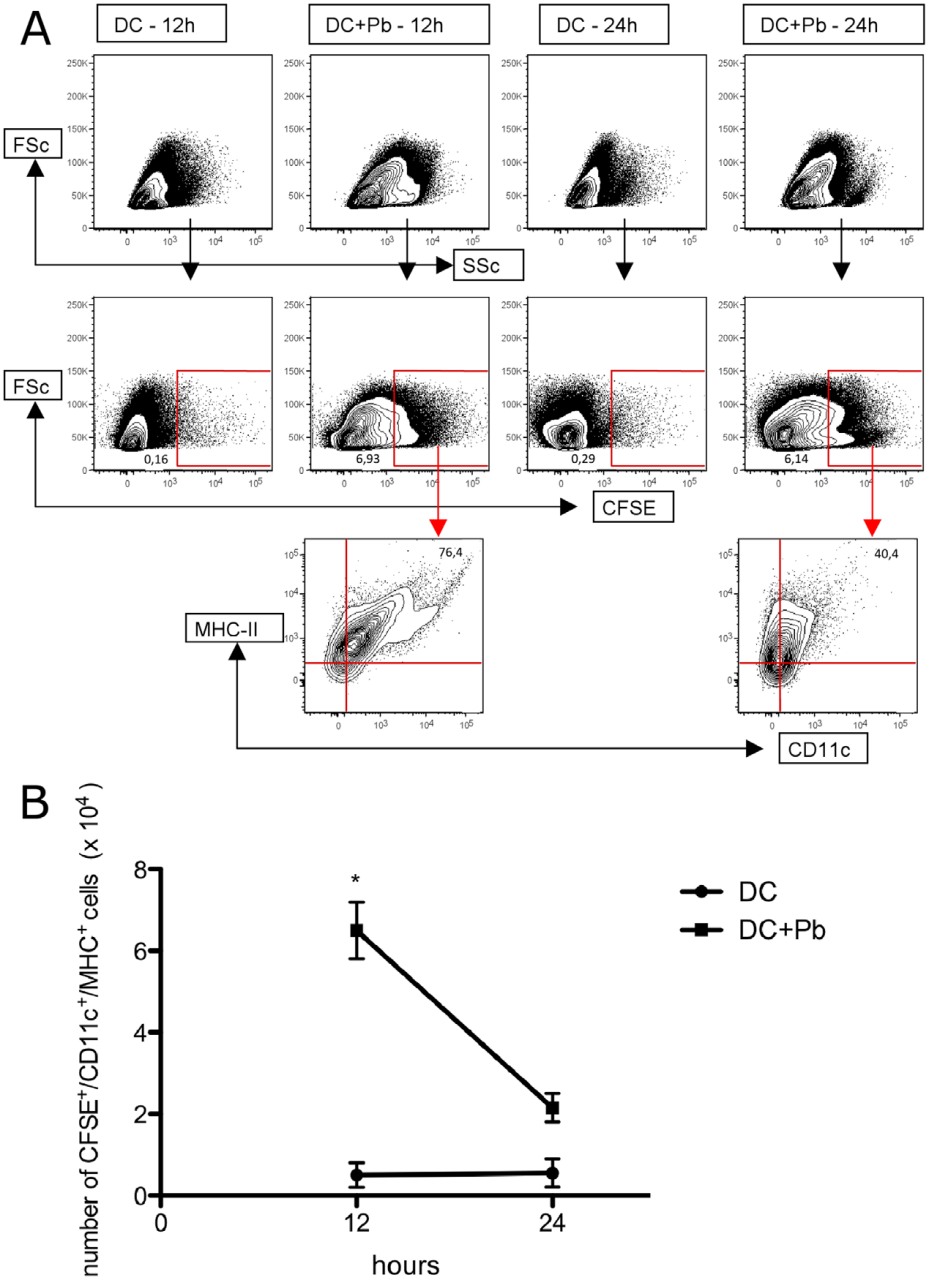
Migration of BM-DC to the lymph nodes. BM-DC were incubated with Pb labeling with CFSE and injected into the lung. After 12 and 24 hrs the lymph nodes cells were analyzed by citometry. A-Figure representative of 3 different experiments, where the gate represent positive cells, determined by FMO, as described in the M&M. The numbers represent the percentage of positive cells. B- At 12 and 24 hrs after Pb infection, the absolute numbers of CFSE+/CD11c+ were determined in the lymph nodes. Error bars represent the SEM. *p<0.05 when compared with only DC.

Naïve T cell priming occurs in lymph nodes draining from the site of infection. To study the capability of DC to migrate to the lung-draining mediastinal lymph nodes, we labeled BM-DC with CFSE, incubated with *Pb* and administered the solution intratracheally to mice. Our results showed an increase of CD11+/CFSE+ in the lymph nodes 12 hrs after administration of fungus, but after 24 hrs the number decreased to control levels (Figure 2B). Only CD11+CFSE+ cells incubated with *Pb* were found in the lymph nodes (Figure 2A). These cells also expressed mature phenotypic markers (MHC-II ^high^). These results demonstrate that BM-DC, in the presence of *Pb,* migrate to draining lymph nodes.

**Figure 3:**
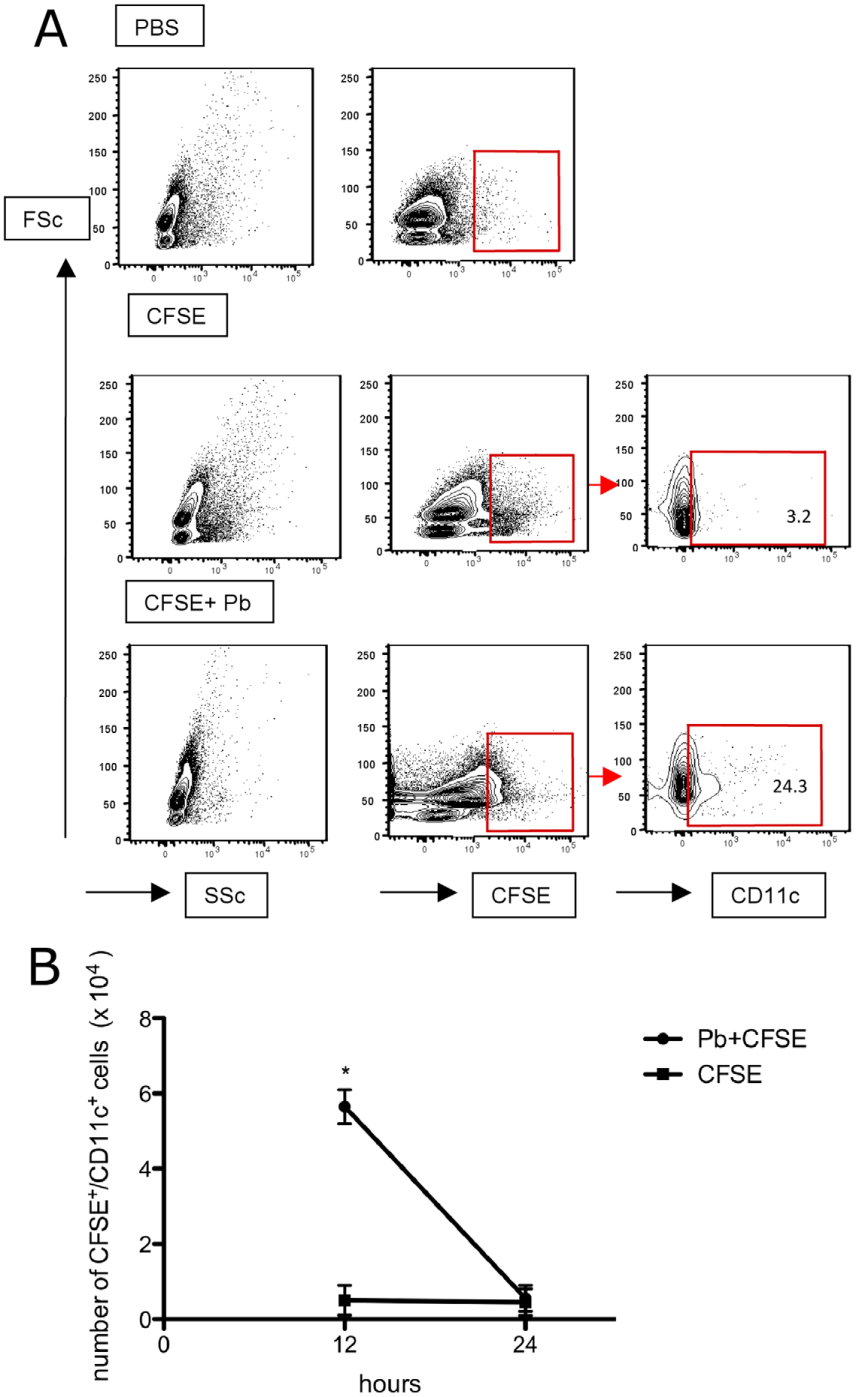
Migration of lung DC to the lympho nodes. CFSE and Pb were injected into the lung and after 12 and 24 hrs the reginal lympho nodes cells were analyzed to the presence of CFSE+/CD11c+ by citometry. A-Figure representative of 3 different experiments where the gate represent positive cells, determined by FMO, as described in the M&M. The numbers represent the percentage of positive cells. B- At 12 and 24 hrs after CFSE and Pb infection, the absolute numbers of CFSE+/CD11c+ were determined in the lympho nodes. Error bars represent the SEM. *p<0.05 when compared with only CFSE.

In addition, we analyzed the capacity of lung DC to migrate to the lung-draining mediastinal lymph nodes. Mice were injected (i.t.) with CFSE and *Pb*. This procedure enabled us to track DC migrating from the lungs to the mediastinal lymph nodes during *Pb* infection. In addition, flow cytometry allowed for the separation of migrating DC and resident DC taken from the lymph nodes. CFSE treatment alone did not induce significant DC migration as demonstrated by the minimal number of CFSE-labeled cells in the mediastinal lymph nodes after infection. After intratracheal infection with *Pb,* the CFSE-labeled cells observed in the mediastinal lymph nodes were predominantly CD11c cells (Figure 3A). It was observed CFSE/CD11c+ cell at 12 hrs after *Pb* infection, and after 24 hrs these cells decreased to control levels (Figure 3B). These results show that lung DC have the capability to migrate to the draining lymph nodes in the presence of *Pb*.

### DC transport yeast of Pb from the airways to the thoracic lymph nodes

**Figure 4:**
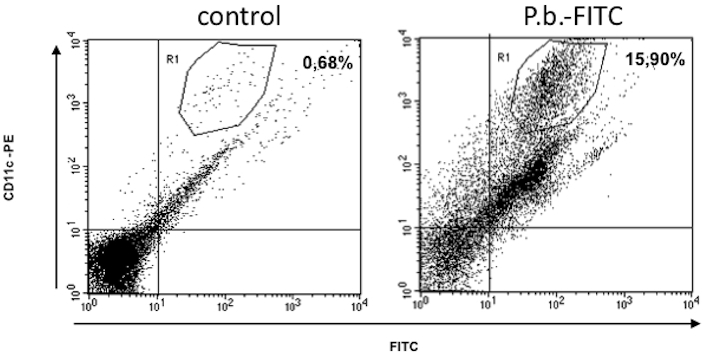
DC transport yeast form of *Pb* to draining lymph nodes. Yeast form of Pb were labeling with FITC as described in the M&M. After labeling, 1×10^6^ *Pb* were injected intratracheally. After 12 h, the lymph node cells were removed and analyzed by cytometry. Animals injected with FITC only were used as controls. The Figure is representative of 3 different experiments. The number represent the percentage of positive cells.

Recently, our group demonstrated that lung DC phagocytose *Pb* yeast *in vivo* [18]. Moreover, in this study, we have shown that lung DC migrate to the lymph nodes. Here, we address the question of whether pulmonary DC, after phagocytosis of the fungus, transport *Pb* yeast from the airways to the lymph nodes. To this end, FITC-labeled yeast cells were i.t. injected, and the FITC-positive DC were enumerated in lymph nodes by FACS analysis. Our results showed that CD11c^+^FITC^+^ cells appeared in the thoracic lymph nodes 12 hrs after infection. These results indicate that pulmonary DC transport *Pb yeast* to the draining lymph nodes (Figure 4).

### Lymph node T-CD4 cell activation by DC

As demonstrated above, DC phagocytose the yeast form of *Pb* and migrate to the lymph nodes. Given these results, DC were considered likely to contribute to the induction of T cell responses.

**Figure 5:**
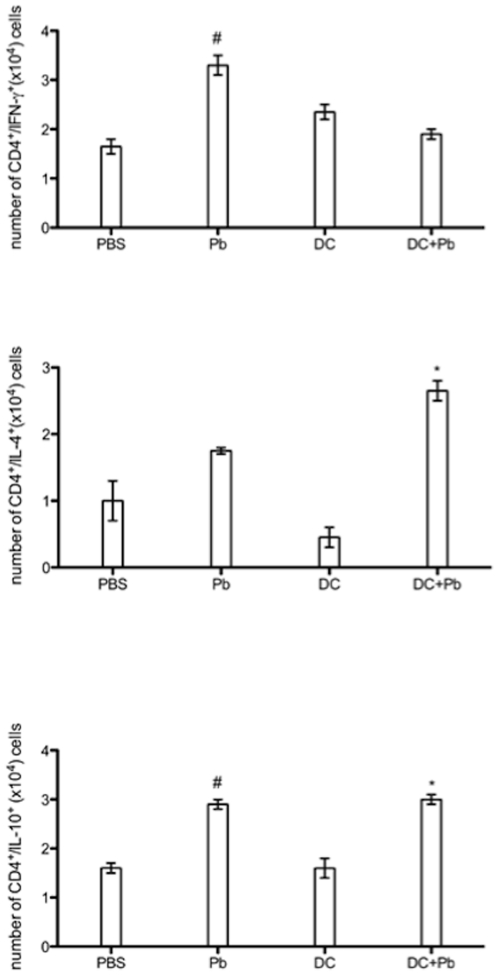
Lymph node T-CD4 cell activation by DC. *A*nimals were injected with *Pb* or *Pb*-pulsed BM-DC, and intracellular cytokines from T cells present in the lymph nodes were investigated. The data represent the mean ± SEM of the results from 3 independent experiments. Error bars represent the standard errors of the means (SEM). *p<0.05 when compared between DC vs. DC+Pb infection or #p<0.05 when compared between PBS vs. Pb infection.

To investigate which CD4 T cell subtypes could be activated by DC, animals were injected with *Pb* or *Pb*-pulsed BM-DC, and intracellular cytokines from T cells present in the lymph nodes were investigated. Our results showed that animals infected with *Pb* induced a mixed pattern of CD4 T cell cytokines, compatible with a Th1/Th2 response. However, when *Pb*-pulsed DC was injected, a Th2 response was observed in the draining lymph node (Figure 5).

## Discussion

PCM starts with inhalation of the fungus; thus, lung cells such as DC are part of the first line of defense against this microorganism. Migration of DC to the lymph nodes is the first step in initiating T cell responses. DC are highly efficient antigen-presenting cells that are central to the induction and regulation of most adaptive immune responses. Their specialized capabilities of acquiring, processing, retaining, and finally presenting peptides on major histocompatibility complex (MHC) molecules are critical properties that account in part for their major role in antigen presentation [19]. Unlike other antigen-presenting cells, DCs are specialized for homing efficiently to the T cell zones of lymphoid organs, allowing for optimal interactions with T lymphocytes. DC migratory capacity distinguishes them from macrophages. Therefore, we postulated that DC, after interaction with *Pb*, could migrate to the draining lymph nodes and activate CD4 T-cells.

In this study, we showed that after *Pb* Infection, an important modification of DC receptor expression occurred. We observed an increased expression of CCR7 and CD103 on lung DC after infection, as well as MHC-II. Several studies have shown that pathogen recognition through PRRs activates DC leading to an increase in DC expression of the chemokine receptors CCR7 and CD103 [20] [21] [11]. The increased expression enables DC to migrate from the site of infection to the secondary lymphoid tissues. Therefore, these results could indicate that DC are capable of migration after interacting with *Pb*. Next, we infected mice with *Pb*-pulsed BM-DC. Our results showed that DC pulsed with *Pb* migrate to the lymph nodes. CFSE+ DC migrate to the lymph nodes, therein coming into contact with naive T cells [11], and this interaction results in the proliferation of naive T cells. Our results showed that *Pb*-pulsed BM-DC induce a Th2-like response when compared to infection with only *Pb*. These results demonstrate the low capability of BM-DC to induce a Th1 response in the presence of *Pb*. Recently, our group showed that BM-DC, in the presence of *Pb,* decreased production of IL-12, a cytokine important for the induction of Th1 [22].

However, it is known that BM-DC and lung DC have differences in response, so it was necessary to test the *in vivo* capacity of lung DC to migrate to lymph nodes following *Pb* infection. Recently, we showed that lung DC can phagocytose the yeast form of *Pb in vivo.* The results of this study showed that lung DC migrate and transport the yeast form of *Pb* to lymph nodes. Migration of lung DC could represent an important mechanism of pathogenesis during PCM infection. The lung DC could act as Trojan horses for *Pb* and, depending on the circumstances, influence disease susceptibility. However, this hypothesis requires more study. In other model of fungal infection it was demostrated that interactions between DC and *Crytococcus neoformans* alter DC antigen-presenting functions and modulate resultant T-cell responses in vitro [23]. Moreover, it was shown that after cryptococcal infection in vivo, DC migrate to thoracic lymph nodes and active T-cell to produce cytokine [24] [25]. Conversely, some pathogens are known to use mechanisms to arrest DC migration to protect themselves from the generation of potent adaptive immune responses. Schistosomal parasites produce prostaglandin D2 to inhibit DC migration to lymph nodes after infection through skin [26], and microbial antigens derived from *Borrelia garinii*, the causative agent of chronic Lyme disease, significantly downregulate CD38 and CCR7 expression in DC, thereby hampering their migratory capability [27].

Elucidating the mechanisms of regulation and coordination of DC migration by pathogen-derived signals requires more study. An understanding of the signals that regulate DC migration could allow for the development of methods to induce efficient T-cell activation that would aid in the control of PCM infection.
